# Physiological traits contribute to growth and adaptation of Mexican maize landraces

**DOI:** 10.1371/journal.pone.0290815

**Published:** 2024-02-01

**Authors:** Brian A. Pace, Hugo R. Perales, Noelymar Gonzalez-Maldonado, Kristin L. Mercer

**Affiliations:** 1 Department of Horticulture and Crop Science, The Ohio State University, Columbus, Ohio, United States of America; 2 Department of Plant Pathology, The Ohio State University, Columbus, Ohio, United States of America; 3 Department of Agroecology, El Colegio de la Frontera Sur, San Cristóbal de Las Casas, Chiapas, Mexico; 4 Department of Land, Air and Water Resources, University of California, Davis, California, United States of America; Central Research Institute for Dryland Agriculture, INDIA

## Abstract

Local adaptation of populations results from an interplay between their environment and genetics. If functional trait variation influences plant performance, populations can adapt to their local environment. However, populations may also respond plastically to environmental challenges, altering phenotype without shifting allele frequencies. The level of local adaptation in crop landraces and their capacity for plasticity in response to environmental change may predict their continued utility to farmers facing climate change. Yet we understand little about how physiological traits potentially underlying local adaptation of cultivars influence fitness. Farmers in Mexico—the crop center of origin for maize—manage and rely upon a high diversity of landraces. We studied maize grown in Chiapas, Mexico, where strong elevational gradients cover a relatively small geographic area. We reciprocally transplanted 12 populations sourced from three elevational zones (600, 1550 and 2150 m) back into those elevations for two years using a modified split-split plot design to model effects of environment, genetics, and their interaction. We studied physiological and growth traits, including photosynthetic rate, stomatal conductance, stomatal density, relative growth rate (RGR), and seed production. Maize fitness showed indications of local adaptation with highland and midland types performing poorly at warmer lowland locations, though patterns depended on the year. Several physiological traits, including stomatal conductance, were affected by G x E interactions, some of which indicated non-adaptive plastic responses with potential fitness implications. We discerned a significant positive relationship between fitness and relative growth rate. Growth rates in highland landraces were outperformed by midland and lowland landraces grown in high temperature, lowland garden. Lowland landrace stomatal conductance was diminished compared to that of highland landraces in the cooler highland garden. Thus, both adaptive and non-adaptive physiological responses of maize landraces in southern Mexico may have implications for fitness, as well as responses to climate change.

## Introduction

In plants, functional traits such as growth, metabolic, physiological, and morphological traits can influence fitness [1, 2]. When fitness is affected by the environment or other factors (and if adequate genetic variation is present), rapid evolution may ensue if selection is strong enough to overcome gene flow [3–5]. Selection pressures vary across geographies and can act on functional plant traits as pivot points in the evolution of local adaptation [6]. Yet, many plant traits also respond plastically to variable environments [7–9]. Thus, as we consider how plant populations may respond to environmental variation, including climate change, we must consider both these evolutionary and ecological dimensions governing plant phenotype [10].

Evolutionary trade-offs over space and time can result in genetic differentiation among populations for physiological traits—an important factor in determining stress resistance in plant populations across environmental clines. For instance, selection can act on growth rates such that growth varies both across and within plant taxa, as well as across geographic (e.g. regional, local) scales [8, 11]. Likewise, photosynthesis can be responsive to local selection pressures (albeit in a more constrained manner). Elevation affects temperature, as well as light intensity (and photon density), so plants from higher elevations may have evolved under cooler, brighter conditions than their lower elevation counterparts, potentially affecting, for example, plant pigment production, stomatal phenotype, and growth rate [10, 12]. Aspects of photosynthesis are also plastically responsive to local environmental change, particularly water availability and temperature [13]. Other traits may respond to environmental stress primarily via plasticity. For example, temperature can play a major role in plant growth by affecting primary metabolism of carbon fixation and carbon loss [14, 15] through rapid modification of both leaf photosynthesis and respiration rate [16–18].

Natural plant populations are well studied for their local adaptation and physiological responses to environmental change (e.g. [19, 20])—processes which have become more important to understand as climate change progresses. However, crop populations are also affected by changing conditions and may or may not be able to respond plastically to all aspects of a changing climate [21]. In fact, anthropogenic climate change has already caused global yield declines since 1980 [22] and the tropics and subtropics are likely to be disproportionately affected in the future [23–25]. Thus, at-risk populations include landraces (or traditional cultivars) maintained by farmers in centers of crop origin. Landraces constitute an important aspect of global crop genetic resources, and their diversity has been shown to be continually evolving—including in response to climate change [21, 26]. For instance, Vigouroux et al., [27] found that pearl millet flowering times adapted to reductions in rainfall over a 40-year interval. In fact, past responses of crops to climatic and geographic variation have shaped the distribution of landraces, which can correspond to broad climatic variation, such as temperature and annual rainfall [28, 29]. Farmer selection on cultivars elicits profound plant performance response, particularly when the desired plant product is the seed or grain (although these are not the only variables farmers care about—see Bellon [30]. In these cases, yield of grain per unit area is a focus which can be seen as a component of plant fitness (or fitness traits), along with survival to reproduction.

Research on maize in Mexico has now shown that crop landraces can be locally adapted and that there is genetic and functional trait differentiation across elevations [31, 32]. Maize locally adapted to elevational gradients [31, 33, 34] also shows elevation-specific allele frequencies [35] and differential gene expression, including for biotic and abiotic stress tolerance loci [36, 37]. But what functional traits govern that differentiation? Several growth, phenological, and physiological traits have been shown to differ between highland and lowland maize races. Thus, local adaptation may result from the influence of one or a combination of functional traits on fitness compared to nonlocal types. Specifically, under low temperatures, highland landraces emerge more completely, their seedlings grow faster, they maintain higher photosynthesis, and have a shorter anthesis-silking interval in cool temperatures compared to lowland landraces [38–40]. Such apparent local adaptation may benefit current productivity but could hamper yields as climate changes. We require greater understanding of the underlying traits—especially physiological ones—that govern these patterns of adaptation, their plastic responses to environment, and of the degree to which traits relate to fitness components. Growth and stomatal traits provide a view into these patterns, both for their ablity to respond plastically and for their capacity to become fixed for environmentally optimal phenotypes.

Here, we present data from two years of experimental field work with maize using reciprocally planted landraces in common gardens along an elevational gradient. We took measurements of gas exchange, growth, and fitness traits under field conditions to clarify patterns of local adaptation and plasticity, while also identifying traits with relationships to fitness. We had several objectives:

1. Discern patterns of local adaptation of landraces along an elevational gradient.
2. Explore patterns of plasticity in growth and physiological traits in maize landraces across an elevational gradient.
3. Determine the degree to which physiological traits relate to seed production.

We predicted that locally adapted plants would outperform nonlocal types. This work aims to provide insight into the physiological mechanisms which may underlie and contribute to local adaptation and phenotypic plasticity in response to changing environmental conditions. Aspects of this work model future conditions for the region predicted with climate change.

## Materials and methods

### Study system and location

Maize was domesticated 9000 years ago in southern Mexico, with recent work demonstrating further domestication in South America [41, 42]. Southern Mexico boasts high levels of genetic diversity [43]. Maize races are distributed according to regional climate [28], with farmer selection practices maintaining these phenotypic distinctions [30]. While maize can be grown in a range of environmental conditions, adaptation to narrow temperature ranges may result in higher production, as cool and warm temperature adaptation may be mutually exclusive [44].

Chiapas, one of the southernmost states in Mexico, hosts large topographical variation in a small geographic area, making it an ideal site for a study of how elevation has shaped maize genetic variation. Maize is cultivated there from 0 to 2600 m in mostly rain-fed systems where farmers save seed from their landraces year after year. Strong elevation gradients co-occur with variation in rainfall, soil type, irradiance, and temperature, which may contribute to the high maize diversity in Chiapas [45]. Along one elevational transect in Chiapas from the Central Highlands south towards the Guatemalan border, rainfall in the rainy season is similar across elevations, although evaporation to precipitation ratios tend to increase at low elevations (S1 Table). Average temperatures drop by roughly 5°C per 1000 m (S1 Table). Thus, maize landraces grown across a varied landscape provide an excellent study system to investigate plasticity and adaptation, as well as traits that underlie that adaptation.

Maize landraces in Chiapas are grown during the wet season. However, across the elevational and environmental gradient represented by our transect, the length and timing of growing season varies. In the highland environment, colder temperatures result in a more extended growing season; maize may be planted between February and mid-May and harvested between mid-November and December, in part because many farmers do not harvest their ears all at once (H. Perales, personal observation). Highland maize is sometimes even planted prior to the initiation of the rains and can have a nine-to-ten-month life cycle (seed to seed). By contrast, in the lowlands, farmers tend to plant their shorter life cycle crop between May and mid-July (i.e., later than in the highlands) and harvest between late October and early December (i.e., earlier than in the highlands) (H. Perales, personal observation).

### Genetic materials

In 2009, we gathered a set of collections of open-pollinated maize landraces within 50 m of three target elevational levels (600 m, 1550 m, and 2050 m). At each elevational level, we gathered 50 whole ears from each of three farmers in each of three communities. We collected only seed from landraces with at least 10 years of cultivation history under that same farmer. As this work was conducted in Chiapas in collaboration with El Colegio de la Frontera Sur (ECOSUR), no permits for maize collection or field experiments (below) were required. We consulted with appropriate village authorities who authorized our approaching farmers for seed and field sites. We bulked seed from the ears we collected from a farmer to create individual populations. Of the 27 populations we collected (described further in Mercer and Perales, 2019), we randomly selected a subset of four from each elevational level (hereafter ‘elevation type’) for this work, for a total of 12 populations (S1 Table). All 12 maize landrace populations from the 2009 collections were used in the 2011 (year 1) and 2012 (year 2) reciprocal common gardens. Populations from each elevation are considered random representatives of that elevation; microclimates may vary among them, in terms of their soils, temperature, and rainfall of origin.

### Common gardens

**Fig 1:**
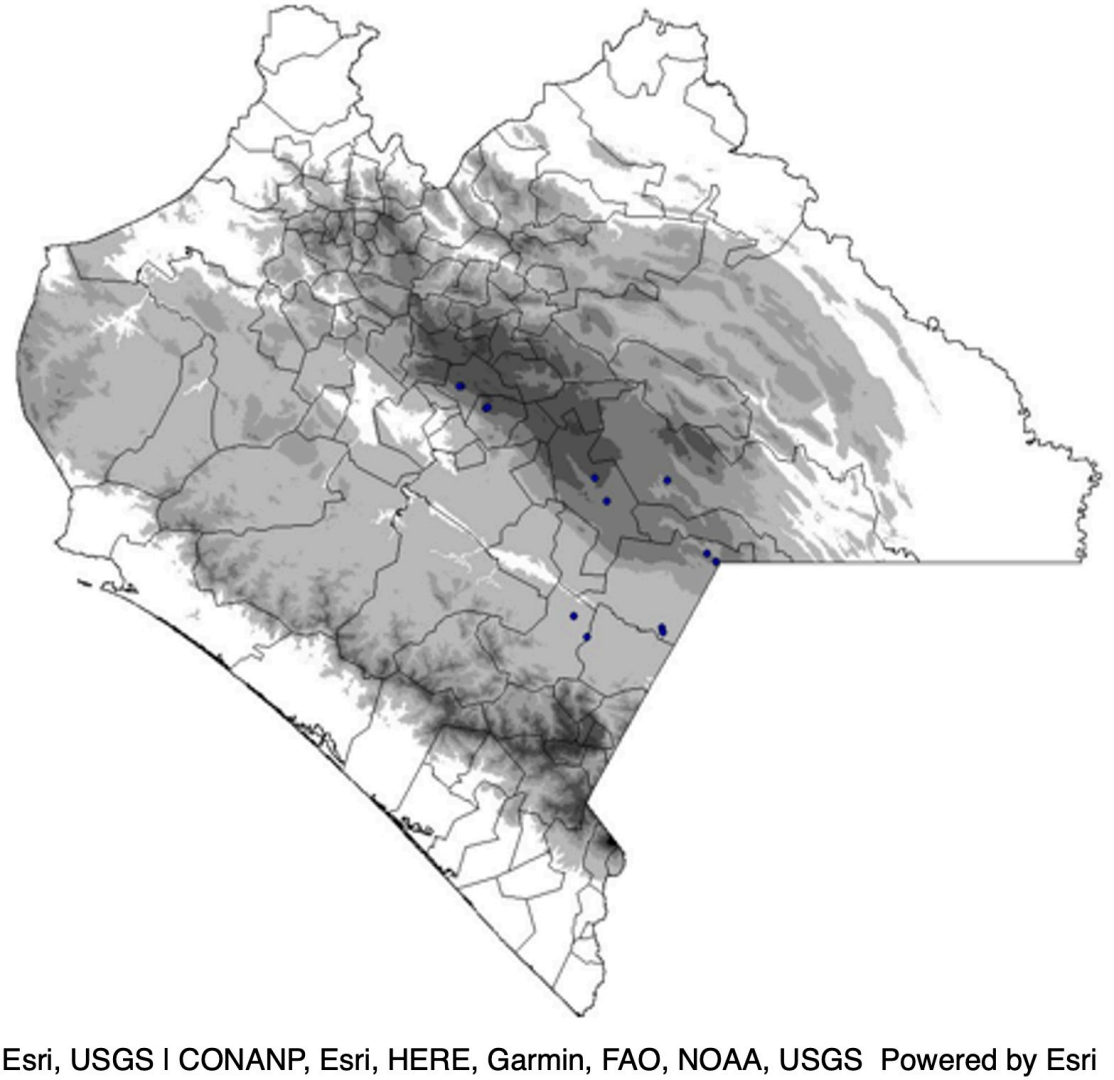
Chiapas, Mexico elevation where darker areas show progressively higher elevation zones. Dots mark locations of communities where maize populations were collected. Stars mark the locations of common gardens. Map by H.R. Perales.

In years 1 and 2, we sowed three reciprocal transplant common gardens along the same elevational transect in Chiapas from which the maize populations were collected to create a synchronic space-for-time design [34, 46] (Fig 1). In each garden, we planted all populations at a date within the normal planting window for each elevation (see above and S2 Table). In both years, the highland garden was located in Chichihuistán (2056 m, 16°35’56.06" N 92°33’41.06" W) and the midland garden was located in La Independencia (1523m, 16°14’30.02” N 92°00’46.44” W). Distinct locations were used for the lowland common garden in year 1 and year 2 –Frontera Comalapa (577 m, 15°50’37.68” N 91°59’56.97 W) and Quespala (600 m, long. 15°81’50.00” N 91°94’77.78” W), respectively–due to unavailability of the original site.

At each field site, we set up the field experiment to elucidate effects of maize origin and timing of harvest for biomass traits (see below). We employed a modified split-split plot design with blocks divided into three main plots, each planted with an elevation type. In year 1, we planted four blocks where elevation type main plots were divided into five aboveground biomass harvest subplots, and subplots were divided into population sub-subplots. By contrast, in year 2, we planted three blocks where elevation type was the main plot, population was the subplot factor, and harvest date was the sub-subplot factor. We created harvest subplots and sub-subplots to allow for multiple destructive biomass harvests throughout the growing season. This change of subplot identity across years was made for ease of harvest. We grouped populations together by elevation type because the different phenology or average heights of plants from different elevational types might have affected neighboring plants in our small subplots (e.g. edge shading). One row of seven (year 2) or two rows of four (year 1) experimental plants made up each sub-subplot and were sown in the traditional fashion, with three seeds per planting location (or *mata*) and one meter between *matas* and rows. Seedlings were thinned to two plants by removing the smallest seedling to maximize chances of having at least two healthy plants for biomass harvests and ear collection. Thus, in year 1 each sub-subplot had 12 *matas* while in year 2 there were 28 *matas* per sub-subplot. We planted border *matas* to surround sub- and main plots to reduce effects of destructive sampling on neighboring subplots and of edge effects, more generally. Border *matas* consisted of a random combination of all landraces of the designated elevation type to avoid edge effects on experimental plants.

### Data collected

We collected data on physiological, growth, and (seed) fitness traits under field conditions. We collected physiological data only in year 1, but measured relative growth rate (RGR) and fitness components in both years. When measuring physiological traits in year 1, we took leaf impressions early in the season (V3) to assess stomatal characteristics and used an infrared gas analyzer before flowering (V12-14) to measure gas exchange. All leaf-level measurements were conducted on one leaf per experimental plant. From the leaf impressions, we calculated stomata and epidermal cell metrics from both adaxial and abaxial impressions, separately. We made stomata impressions on one mature, fully expanded leaf (V3) from one harvest subplot for each maize population on the adaxial (“top”) and abaxial (“bottom”) central surfaces using a ~2 cm diameter application of film-forming nitrocellulose polymer dissolved in ethyl acetate (i.e., clear nail polish). We then removed the dried nitrocellulose films from leaves using clear tape and affixed them to microscope slides. We collected impressions in the highland and midland gardens only at identical growth phases across gardens. We counted stomata and epidermal cells using a compound microscope 40x objective, a video display (Fisherbrand Micromaster, Waltham, MA, USA), and a Westover TFT-LCD color monitor (Woodinville, Washington, USA), counting all stomata and epidermal cells in the viewing frame (0.0793 mm^2^). Any cells or stomata located on the edge of the viewing field were counted only when 50% or more of the structure was visible. Stomata (SD) and epidermal cells densities (ED) (per cm^2^) were calculated and used to estimate stomata per leaf (SL). Stomata index (SI) was calculated as:

$$\text{SI}=\left[\#\text{stomata}/\left(\#\text{stomata}+\text{epidermal}\;\text{cells}\right)\right]\;\text{x}\;100$$

We also conducted infrared gas analysis at all gardens prior to flowering (V13-V14) between the hours of 10 am and 2 pm at identical growth phases across gardens. Dates of measurements were chosen using flowering data from the previous year to occur shortly before flowering at each garden. For each garden, we took measurements in one day on the youngest fully expanded mature sun leaf for each experimental plant (one per *mata*) using an LC*i* infrared gas analyzer (ADC BioScientific Ltd., UK). At all gardens, we collected instantaneous measurements of leaf temperature, photosynthetic rate (A), transpiration (T), stomata conductance (gs), and leaf photosynthetic photon flux density (PPFD) for three randomly selected plants per population from a single harvest sub-subplot (S3 Table). Water Use Efficiency metrics were also derived from these data, with instantaneous (WUE_ins_) and intrinsic (WUE_int_) values possible (S2 Table). Stomatal resistance describes the rate of CO_2_ entry into the leaf, while stomatal conductance describes the passage of H_2_O out of the leaf via evaporation [47]. Because the LC*i* Leaf Chamber Analysis System uses ambient light rather than an independent light source, we standardized photosynthetic rate to available light (A/PPFD) as light use efficiency (LUE) (S3 Table).

We calculated relative growth rate (RGR) for each garden location and elevation of origin within location by block. At each harvest, we noted vegetative leaf stage and destructively sampled one randomly selected plant per *mata*. We collected total aboveground biomass, split the plant down the stalk, and dehydrated it (along with ears at final harvest) in a custom-built biomass drier at 65°C for at least 72 hours (or up to one week with more mature plants). For growth traits, we took destructive aboveground shoot biomass harvests at several dates over the course of the season; the frequency and timing of those measures differed in years 1 and 2, but they were chosen using elevation-specific maize growth stage from previous experiments (H. Perales and K. Mercer, personal observation). While our experiment was designed to accommodate more vegetative biomass harvests times, importantly, we only used two to calculate relative growth rate each year—those taken from early seedling and later vegetative stages. Thus, we will focus our attention on describing those two harvest periods here (there is additional harvest information in S3 Table). Since season length differed at each common garden location, the exact dates of collection and life stage assayed varied across gardens and years. In both years, we destructively sampled early seedling vegetative biomass between V4 and V6. Later vegetative growth was assayed at V6-11 in year 1 and V15-16 in year 2.

For seed fitness traits, we harvested grain from all individual ears on each randomly selected focal plant and assessed each for whether it produced any seed or not (to calculate the probability of reproducing), seed production per individual surviving to reproduce (grain weight per reproductive plant), and grain weight per emerged seedling (calculated as the probability of survival multiplied by the seed weight per emerged seedling).

### Data analysis

We analyzed two categories of generalized linear mixed models in Proc GLIMMIX (SAS, Cary, North Carolina, USA, 2008) using restricted maximum likelihood. We used the first category of model to analyze various response variables: probability to reproduce (binomially distributed, logit link function), grain weight per reproductive plant, grain weight per emerged seedling, RGR, stomatal index, stomatal density, epidermal cell density, and gas exchange traits (S2 Table). As the residuals were strongly skewed, LUE was logit transformed. Each time we ran our first category of model, we aimed to detect main effects of the fixed factors, elevation type and garden location, as well as interactions between them (i.e., elevation type by garden location interaction), a form of genotype by environment interaction, or G x E. Each model also included the random effects of block within common garden, population within elevational type, and the interaction between common garden and population within elevational type. Due to the modified split-split plot nature of our design at each garden, elevational type was tested with the interaction between elevational type and block within common garden. We did not include harvest date in the model because data was collected from only one harvest for each trait; RGR required use of data from two harvests but resulted in a single value being used. We produced least squares means along with Tukey-Kramer mean separations between elevations of origin in each garden. We tested block within garden, population within elevation, and the interaction between garden location and population within elevation of origin as to whether they were greater than zero with a log-likelihood test. We performed all analyses on plot means to alleviate the possibility of pseudoreplication affecting our results.

In our second model, we aimed to illuminate the relationship of growth and physiological traits to grain weight per emerged plant by including continuous traits as predictors of fitness response. These additional predictors were included jointly to allow their covariation to influence slopes of the relationship of each to fitness. This approach emphasizes direct, rather than indirect relationships, potentially indicative of direct selection. To reduce the overall number of predictive variables in the model, we calculated Pearson correlations among plot means for all variables using Proc CORR (SAS, Cary, North Carolina, USA, 2008). Based on the results, we removed from consideration variables where strong correlations existed since strong correlations can erroneously affect model results (S4 Table). All gas exchange traits (S2 Table) were highly correlated with one another. Thus, this second model built on the first one (above), but added RGR, stomatal conductance, and LUE as continuous predictors of grain weight per emerged seedling in year 1; only RGR was added for year 2.

## Results

**Fig 2:**
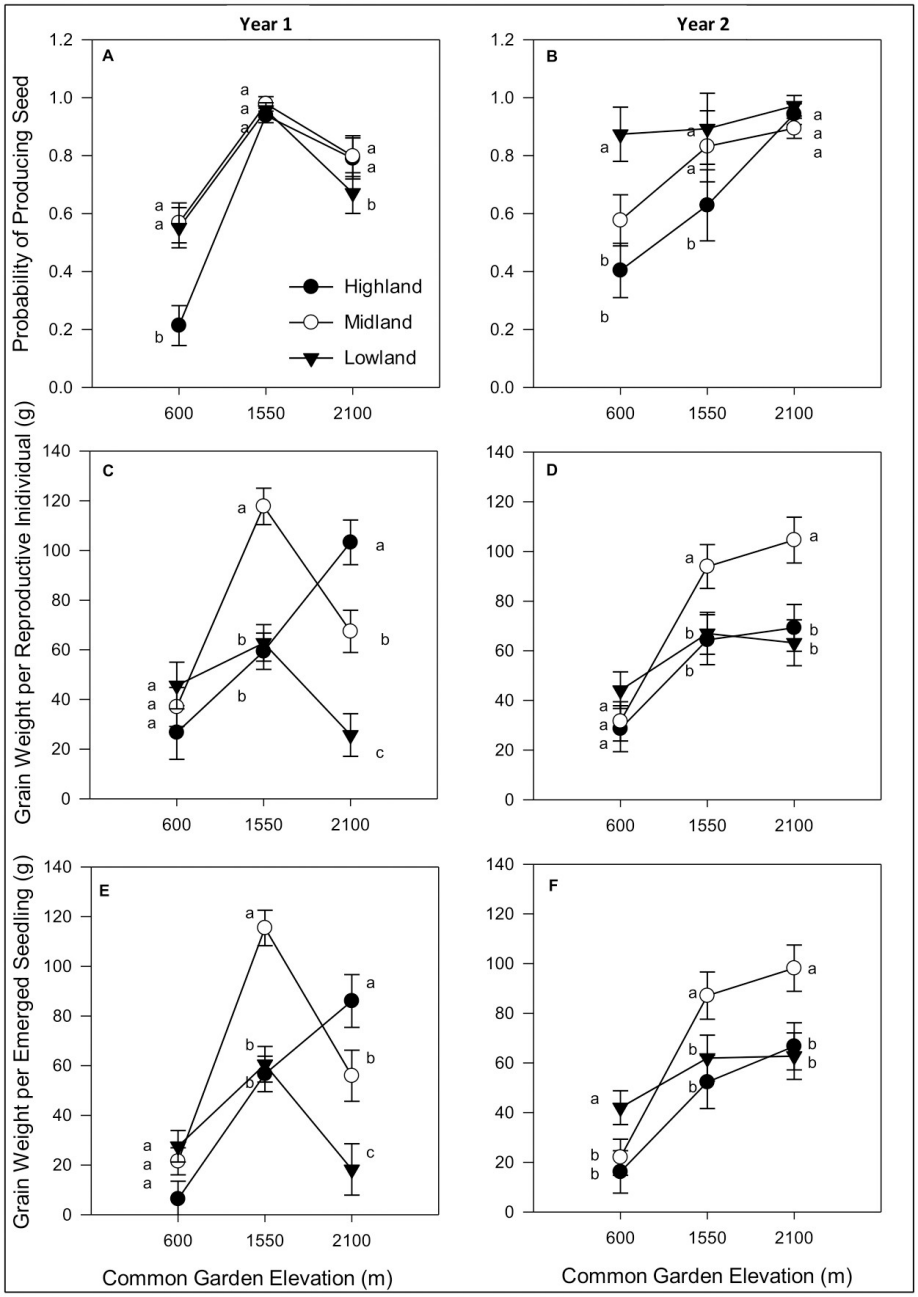
Probability of producing seed by elevation type in all three common gardens in year 1 (**A.)** and year 2 **(B.)**; Grain weight per reproductive plant across all gardens in year 1 (**C.)** and year 2 (**D.**); Total grain weight per emerged seedling, all gardens, year 1 (**E.)** and year 2 (**F.)**. Tukey-Kramer means separation are valid within garden only. Values with the same letter are not statistically different.

We found that fitness components were affected by garden, elevational type, and their interaction, but that responses varied across years and traits. For year 1, there were significant G x E interactions for probability to reproduce (Table 1). While all types were equivalent in the midlands, the highland type had the lowest probability of reproducing in the lowland garden and the lowland type had the lowest probability of reproduction in the highlands (Fig 2A). By contrast, in year 2, only the main effects of garden and elevation affected the probability of reproducing (Table 1), with garden elevation increasing the probability of reproducing and elevation of origin decreasing it (Fig 2B). We saw the most variability in the lowland garden, where lowland types reproduced nearly 100% of the time, while other types had much reduced probabilities of reproducing (Fig 2B). In both years, the probability of reproducing was surprisingly low for the highland types in the lowland garden (approximately 0.20 and 0.40, for years 1 and 2, respectively; Fig 2A and 2B) despite overlapping anthesis-silking intervals.

**Table 1:** Generalized linear mixed models predicting fitness components in years 1 and 2 demonstrating the effect of common garden, elevation of origin and their interaction, as well as block within garden, population within elevation, and garden by population within elevation effects. A. Probability to produce seed (values are logit transformed); B. Grain weight; and C. Grain weight per plant that produced seed. Fixed factor degrees of freedom list values for numerator and denominator df. Random factors were tested with a log likelihood.

**A. Probability to reproduce**		**Year 1**				**Year 2**		
**Fixed Factors**	**DF**		**F**	**P**	**DF**		**F**	**P**
Garden	2, 9		18.81	**0.0006**	2, 6		7.78	**0.0216**
Elevation	2, 9		4.31	**0.0486**	2, 10		4.92	**0.0326**
Garden*Elevation	4, 17		4.61	**0.0105**	4, 12		1.4	0.2908
**Random Factors**	**DF**	**-2RLL**	**ChiSq**	**P**	**DF**	**-2RLL**	**ChiSq**	**P**
Block (garden)	1	-2208.21	3.39	0.0657	1	-154.7	0	1
Pop(elev)	1	-2211.6	0	1	1	-154.7	0	0.9927
Elev*blk(garden)	1	-753.69	1457.91	**< .0001**	1	-129.92	24.78	**< .0001**
Garden*pop(elev)	1	-2202.4	9.2	**0.0024**	1	-49.97	104.73	**< .0001**
**B. Total grain weight per emerged seedling**								
**Fixed Factors**	**DF**		**F**	**P**	**DF**		**F**	**P**
Garden	2, 9		15.29	**0.0013**	2, 7		26.15	**0.0011**
Elevation	2, 9		6.75	**0.0162**	2, 10		3.46	0.072
Garden*Elev	4, 16		20.33	**< .0001**	4, 12		3.39	**0.0447**
**Random Factors**	**DF**	**-2RLL**	**ChiSq**	**P**	**DF**	**-2RLL**	**ChiSq**	**P**
Block(garden)	1	3155.71	0.14	0.7072	1	2469.99	0	1
Pop(elev)	1	3157.4	1.83	0.1764	1	2471.81	1.82	0.177
Elev*blk(garden)	1	3155.57	0	1	1	2469.99	.	1
Garden*pop(elev)	1	3155.57	0	1	1	2470.27	0.29	0.5929
**C. Grain weight per reproductive plant**								
**Fixed Factors**	**DF**		**F**	**P**	**DF**		**F**	**P**
Garden	2, 9		18.36	**0.0007**	2	6	26.33	**0.0011**
Elevation	2, 9		6.63	**0.017**	2	10	3.74	0.0612
Garden*Elev	4, 16		14.19	**< .0001**	4	12	3.27	**0.0495**
**Random Factors**	**DF**	**-2RLL**	**ChiSq**	**P**	**DF**	**-2RLL**	**ChiSq**	**P**
Block(garden)	1	3107.28	1.32	0.2511	1	2464.83	0	1
Pop(elev)	1	3107.38	1.42	0.2338	1	2465.7	0.87	0.3513
Elev*blk(garden)	1	3107.43	1.46	0.2264	1	2464.98	0.15	0.7024
Garden*pop(elev)	1	3105.97	0	1	1	2465.64	0.81	0.3682

Both grain weight per reproductive plant and per emerged seedling were significantly affected by interactions between garden and elevation of origin in both years (Table 1). In year 1, these G x E interactions took the form of highest grain weight per reproductive plant in the midland garden by the midland type with highland and lowland types performing similarly. In the highland garden, the highland type produced the most with midland and lowland types producing progressively less; types performed similarly to one another in the lowland garden (Fig 2C). For year 2, grain weight per reproductive plant was lowest in the lowland garden, with all types producing similarly little grain. Midland types outperformed the lowland and highland types in the midland garden and, surprisingly, in the highland garden too (Fig 2D). When the probability of reproducing was factored into grain weight as grain weight per emerged seedling, we saw similar patterns (Fig 2E and 2F).

**Fig 3:**
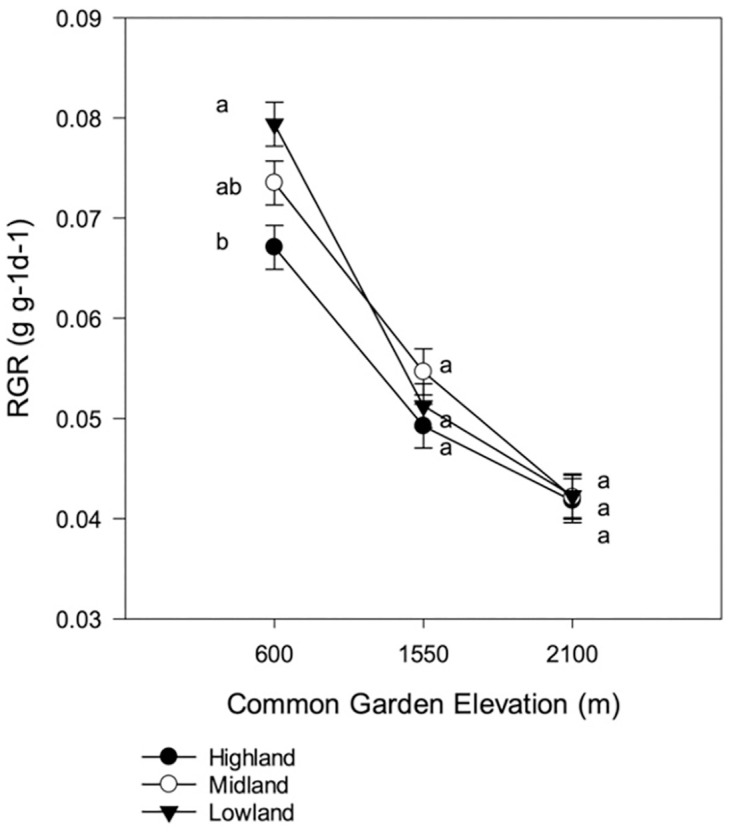
Relative growth rate in year 2 expressed as grams increase per aboveground dry biomass per day. Tukey-Kramer means separation are valid within garden only. Values with the same letter are not statistically different.

**Fig 4:**
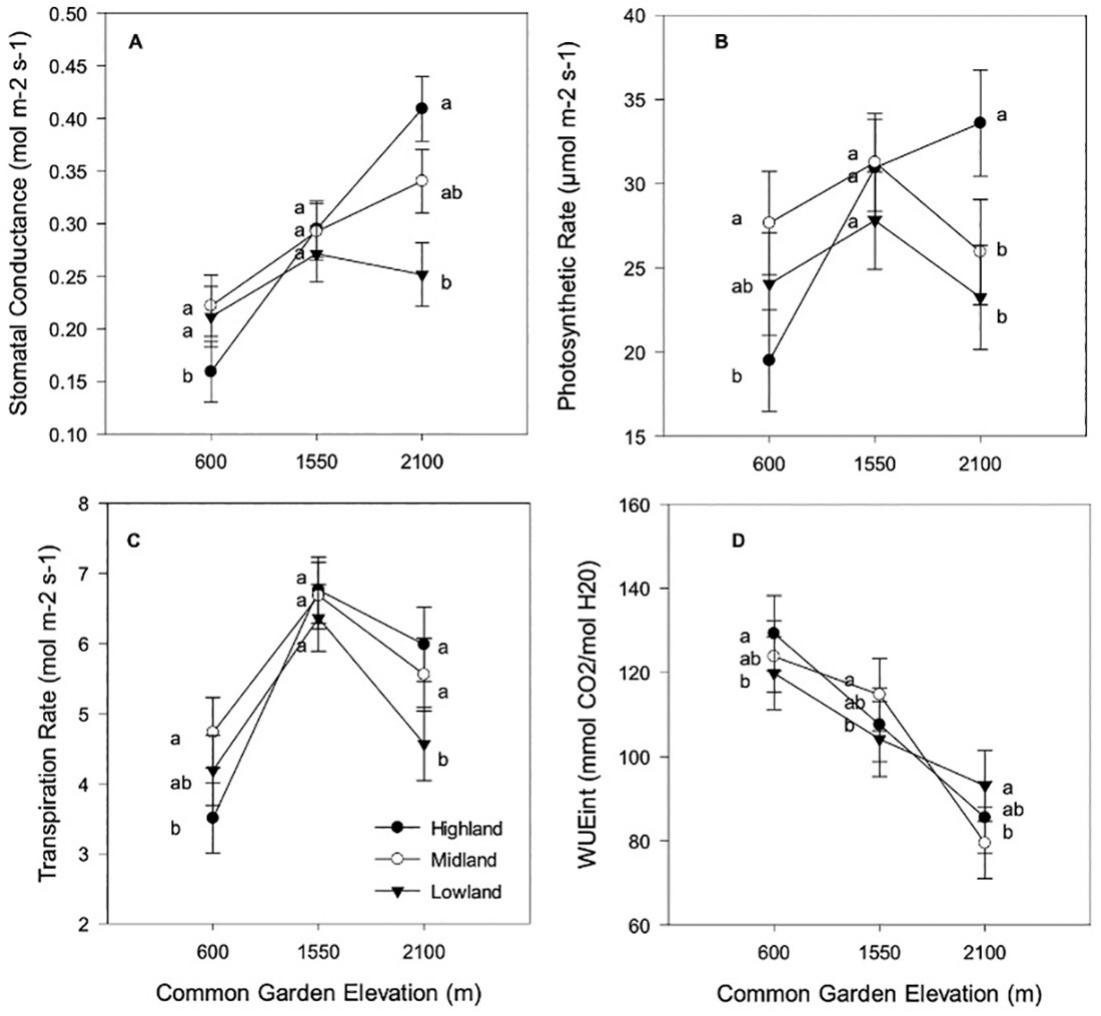
Gas exchange traits for all maize types at all gardens: **A.** Stomatal conductance; **B.** Photosynthetic rate; **C.** Transpiration rate; **D.** Intrinsic water use efficiency. Tukey-Kramer means separation is valid within common garden location only. Values sharing the same letter are not significantly different. Highland types are marked with filled circles, Midland with empty circles, and Lowland types are indicated with filled triangles.

For our physiological traits, garden location had the largest effect (Tables 2 and 3). RGR and WUE_int_ declined with increasing elevation: RGR (year 1) was 0.0453 (s.e. 0.00080) g g^-1^d^-1^ in the lowland garden and 0.0363 (s.e. 0.00065) and 0.0379 (s.e. 0.00080) g g^-1^d^-1^ in the midland and highland gardens, respectively (see Fig 3 for RGR (year 2) and Fig 4D for WUE_int_). Like RGR and WUE_int_, LUE and transpiration were significantly affected by garden location (Table 3), but were highest in the midland garden (LUE: lowland garden: 0.66 ± 0.061, midland garden: 0.9 ± 0.051, highland garden: 0.72 ± 0.045; see Fig 4C for Transpiration).

**Table 2:** Generalized linear mixed models predicting differences relative growth rates (RGR) for years 1 and 2 at all gardens. Fixed factor degrees of freedom list values for numerator and denominator df. Random factors were tested with a log likelihood.

RGR	Year 1				Year 2			
**Fixed Factors**	**DF**		**F**	**P**	**DF**		**F**	**P**
Garden	2, 9		40.85	**< .0001**	2, 6		161.91	**< .0001**
Elevation	2, 9		0.05	0.9496	2, 9		4.21	0.0513
Garden*Elev	4, 16		1.95	0.1513	4, 12		2.58	0.0912
**Random Factors**	**DF**	**-2RLL**	**ChiSq**	**P**	**DF**	**-2RLL**	**ChiSq**	**P**
Block(garden)	1	-761.13	0	1	1	-657.15	0	1
Pop(elev)	1	-761.13	0	1	1	-657.15	0.01	0.9356
Elev*blk(garden)	1	-761.13	0	1	1	-657.15	0	1
Garden*pop(elev)	1	-761.11	0.02	0.8841	1	-657.15	0	1

**Table 3:** Generalized linear mixed models predicting gas exchange traits measured in year 1. Fixed factor degrees of freedom list values for numerator and denominator df. We tested random factors with a log likelihood test. A: photosynthetic rate; gs: stomata conductance; T: leaf transpiration; LUE: light use efficiency (logit transformed); WUEi: intrinsic water use efficiency. Random factors were tested with a log likelihood.

	Fixed Factors	DF	F	P	Random Factors	DF	-2RLL	ChiSq	P
**A**	Block	3, 6	1.26	0.3697	Block(garden)	1	1456.84	2.22	0.1363
	Garden	2, 6	2.07	0.2069	Pop(elev)	1	1455.96	1.34	0.2463
	Elevation	2, 6	1.41	0.2933	Elev*blk(garden)	1	1463.65	9.03	**0.0027**
	Garden*Elevation	4, 15	3.26	**0.0413**	Garden*pop(elev)	1	1454.62	0	1
**gs**	Block	3,6	1.22	0.3823	Block(garden)	1	-374.52	2.11	0.1464
	Garden	2, 6	10.93	**0.01**	Pop(elev)	1	-374.88	1.75	0.1864
	Elevation	2, 9	2.26	0.1601	Elev*blk(garden)	1	-376.04	0.59	0.4434
	Garden*Elevation	4, 15	5.74	**0.0052**	Garden*pop(elev)	1	-376.62	0	1
**T**	Block	3, 6	0.2	0.8932	Block(garden)	1	767.44	8.15	**0.0043**
	Garden	2, 6	6.43	**0.0322**	Pop(elev)	1	760.72	1.43	0.2316
	Elevation	2, 6	1.51	0.2722	Elev*blk(garden)	1	763.24	3.94	**0.0471**
	Garden*Elevation	4, 15	2.39	0.0975	Garden*pop(elev)	1	759.29	.	1
**LUE**	Block	3, 6	1.15	0.4034	Block(garden)	1	114.34	2.34	0.1257
	Garden	2, 6	32.08	**0.0006**	Pop(elev)	1	112.38	0.38	0.5393
	Elevation	2, 6	1.3	0.3197	Elev*blk(garden)	1	117.03	5.03	**0.0249**
	Garden*Elevation	4, 15	0.4	0.8027	Garden*pop(elev)	1	112	0	1
**WUEi**	Block	3, 6	0.42	0.7444	Block(garden)	1	1940.92	6.34	0.0118
	Garden	2, 6	12.14	**0.0078**	Pop(elev)	1	1934.58	0	1
	Elevation	2, 9	0.11	0.8929	Elev*blk(garden)	1	1934.58	0	1
	Garden*Elevation	4, 15	1.96	0.1523	Garden*pop(elev)	1	1934.68	0.11	0.7455

Interactions between garden and elevation affected photosynthetic rate and stomatal conductance (Table 3). Stomatal conductance showed crossing of reaction norms and changes in rank between elevational types; highland and midland types had highest conductance at the highland garden, while the highland type had the lowest conductance at the lowland garden (Fig 4A). The photosynthetic rate of the highland type was higher than the others in the highland location and was lower than the midland type in the lowest elevation (Fig 4B). The near significant interaction between elevational type and garden for RGR in year 2 (P = 0.0912) indicates that it trended to being higher for lowland types than highland types at the lowland garden (Fig 2). Similarly, a near significant interaction also affected transpiration (P = 0.0975), such that the lowland type trended towards having the lowest transpiration rates in the highland garden and the highland type trended towards having the lowest one in the lowland garden (Fig 4C).

**Fig 5:**
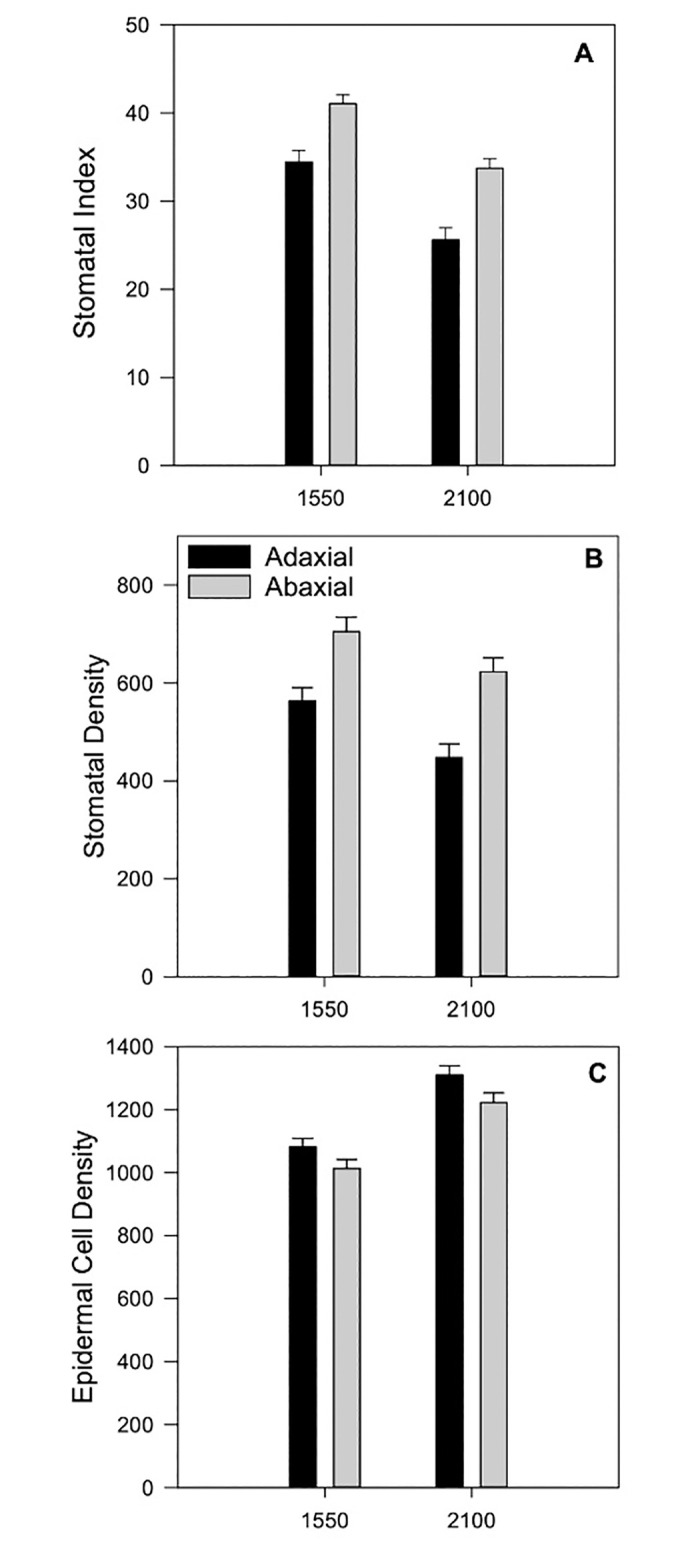
Maize leaf stomata traits by garden elevation and leaf side (abaxial or adaxial) averaged over maize types. **A.** Stomatal index; **B.** Stomatal density; **C.** Epidermal cell density.

Stomatal index, stomatal densities, and epidermal cell densities were significantly influenced by garden location, with stomatal density and index decreasing and epidermal cell density increasing (i.e., reducing cell size) with elevation (Table 4; Fig 5A–5C). Epidermal cell density was also affected by elevation (abaxial side significant, adaxial side nearly significant with P = 0.0521), with lowland maize producing higher densities (i.e., smaller cell size) than midland and highland maize [Adaxial epidermal cell density: lowland maize, 1253.6 cells/cm^2^ (35.69); midland maize, 1219.3 cells/cm^2^ (38.84); highland maize, 1116.3 cells/cm^2^ (35.22); Abaxial epidermal cell density: lowland maize, 1214.1 cells/cm^2^ (33.76); midland maize, 1081.1 cells/cm^2^ (34.04); highland maize, 1059.7 cells/cm^2^ (33.47)]. Epidermal cell densities were nearly significant (adaxial: P = 0.054; abaxial: P = 0.084) for interactions between garden and elevation (Table 4, S1 Fig).

**Table 4:** Generalized linear mixed models predicting stomata impression traits for highland and midland garden only. Fixed factor degrees of freedom list values for numerator and denominator df. Random factors were tested with a log likelihood.

	**Stomata Index Adaxial**				**Stomata Index Abaxial**			
**Fixed Factors**	**DF**		**F**	**P**	**DF**		**F**	**P**
Garden	1, 6		20.9	**0.0038**	1, 6		26.97	**0.002**
Elevation	2,9		0.72	0.5147	2,9		0.13	0.882
Garden*Elev	2, 9		1.09	0.3768	2, 9		0.7	0.5213
**Random Factors**	**DF**	**-2RLL**	**ChiSq**	**P**	**DF**	**-2RLL**	**ChiSq**	**P**
Block(garden)	1	1262.18	6.83	**0.009**	1	1276.34	4.16	**0.0413**
Pop(elev)	1	1255.34	0	1	1	1272.98	0.79	0.3728
Elev*blk(garden)	1	1259.36	4.02	**0.0451**	1	1273.34	1.16	0.281
Garden*pop(elev)	1	1255.34	0	1	1	1272.28	0.1	0.7568
	**Stomata Density Adaxial**				**Stomata Density Abaxial**			
**Fixed Factors**	**DF**		**F**	**P**	**DF**		**F**	**P**
Garden	1, 6		8.78	**0.0252**	1, 6		4.56	0.0767
Elevation	2, 9		2.39	0.1469	2, 9		3.56	0.0724
Garden*Elev	2, 9		1.53	0.2689	2, 9		0.1	0.9056
**Random Factors**	**DF**	**-2LL**	**ChiSq**	**P**	**DF**	**-2LL**	**ChiSq**	**P**
Block(garden)	1	2564.91	13.14	**0.0003**	1	2727.4	7.05	**0.0079**
Pop(elev)	1	2551.82	0.05	0.8158	1	2723.71	3.37	0.0665
Elev*blk(garden)	1	2551.78	0.02	0.888	1	2720.67	0.33	0.5663
Garden*pop(elev)	1	2551.77	0	1	1	2720.34	0	1
	**Epidermal Cell Density Adaxial**				**Epidermal Cell Density Abaxial**			
**Fixed Factors**	**DF**		**F**	**P**	**DF**		**F**	**P**
Garden	1, 6		39.28	**0.0008**	1, 6	6	30.32	**0.0015**
Elevation	2, 9		4.18	0.0521	2, 9	9	7.33	**0.0129**
Garden*Elev	2, 9		4.11	0.054	2, 9	9	3.3	0.084
**Random Factors**	**DF**	**-2LL**	**ChiSq**	**P**	**DF**	**-2LL**	**ChiSq**	**P**
Block(garden)	1	2953.09	0.02	0.8855	1	2875.72	1.11	0.2926
Pop(elev)	1	2954.9	1.83	0.1758	1	2878.17	3.55	0.0594
Elev*blk(garden)	1	2957.34	4.28	**0.0386**	1	2876.91	2.29	0.1298
Garden*pop(elev)	1	2953.07	0	1	1	2874.61	.	1

When assessing relationships between our physiological measures and grain weight per emerged plant in year 1, we found that RGR had a significant positive relationship (β = 2119.94; P = 0.0013), stomatal conductance trended towards a significant positive relationship (β = 51.09; P = 0.0979), and LUE was not related to fitness (Table 5).

**Table 5:** Generalized linear mixed models predicting maize landrace grain weight per emerged seedling jointly from physiological measurements, stomatal conductance (gs), Light Use Efficiency (LUE), relative growth rate (RGR), and experimental factors in year 1. Fixed factor degrees of freedom list values for numerator and denominator df. Random factors are tested with a log likelihood. Continuous predictors include regression coefficients (***β***) quantifying the relationship of each to maize grain weight.

	Year 1				
**Fixed Factors**	**DF**		**F**	**P**	**β (se)**
Garden	2, 9		34.65	**< .0001**	
Elevation	2, 9		5.72	**0.0249**	
Garden*Elevation	4, 13		9.9	**0.0007**	
Gs	1, 32		2.91	0.0979	51.09 (29.97)
LUE	1, 32		0.13	0.7206	7.62 (21.13)
RGR	1, 32		12.46	**0.0013**	2119.94 (600.66)
**Random Factors**	**DF**	**-2RLL**	**ChiSq**	**P**	
Block(garden)	1	710.68	0.08	0.7748	
Pop(elev)	1	711.11	0.51	0.474	
Elev*blk(garden)	1	710.98	0.38	0.5386	
Garden*pop(elev)	1	710.6	0	1	

## Discussion

Our research with maize landraces showed strong evidence for interactions between elevation of origin (G) and garden location (E), demonstrating a G x E interaction affecting all fitness traits except probability to reproduce in year 1. The nature of this interaction was such that most elevational types exhibited some degree of local adaptation in both years. Types from lowland and midland elevations maintained more constant fitness with environmental change than highland types, which might be due to plasticity of traits we measured, such as RGR, and likely others that we did not measure. Some physiological and growth traits displayed clear G x E interactions (e.g., photosynthetic rate and stomatal conductance), though the majority were most responsive to garden elevation. Nevertheless, RGR and, to some degree, stomatal conductance seems to predict fitness (grain weight per reproductive plant) in year 1. Thus, variation in growth and physiological traits may account, in part, for a portion of the underlying differential local adaptation to elevation in these maize landraces. These sources of adaptation may play a role in the adaptive patterns in maize landraces more broadly [31].

### Local adaptation and phenotypic plasticity in physiological traits

A component of local adaptation likely results from the capacity for adaptive plasticity in traits contributing to fitness, which itself constitutes a functional trait [48, 49]. The patterns of local adaptation noted here varied annually (as seen in Mercer and Perales [34]) and were not necessarily symmetrical, in that some types responded more to environmental change than others (as seen in Mercer et al. [33]). Interestingly, the fitness responses we measured here differ somewhat from those found in an experiment conducted the same year and geographical area [34]. In particular, the fitness components measured in neighboring midland garden locations in year 2 were much lower in Mercer and Perales [34]. During that drier than normal season, our plants and theirs were separated by approximately 200–300 m and our plants fared better than those at the other location, particularly for ability to reproduce (see [32]). Perhaps our plots better retained soil moisture (see Grassein et al. [50] for other examples). Thus, fine-grained spatial variation can influence assessments of local adaptation, as well.

Functional polymorphisms contributing to the adaptation across varied landscapes have been observed in plant traits [51, 52]. Physiological and growth traits, such as RGR, have been observed to vary within species across altitudinal gradients [53] and may influence a population’s ability to maintain itself. However, we found greater response of our physiological traits to environment than we did to genetics or G x E interactions. Plasticity of stomatal and epidermal cell densities is well-documented [13]. During leaf development, cells differentiate into either epidermal cells or stomata [54]. Here, stomatal indices and densities plastically decreased with increasing elevation, likely due to decreased temperature stress at higher elevation; higher temperatures typically elicit increased need for transpiration. Our data is consistent with the high plasticity expressed by stomatal index and densities for many species [13]. For instance, in *Pseudoroegneria spicata*, temperature and water availability induced a plastic reduction in abaxial stomatal density and leaf area with reduced stress [55]. By contrast, we found that epidermal cells densities responded to both garden elevation and elevation of origin, indicating that there was a genetic effect of decreased densities in higher elevation maize types in addition to the plasticity in response to environment. Stomatal phenotypes may differ not only in their density and leaf-level location, but in their comparative sensitivity to gas concentrations, temperature variation, and available ground water [11, 56].

Ecophysiological methods can help clarify the mechanisms underlying local adaptation and phenotypic plasticity [11, 49]. Basic gas exchange traits (e.g., stomata conductance, photosynthetic rate) represent fundamental mechanisms of primary plant metabolism that underlie growth and fitness. This work provided a snapshot into the gas exchange strategies employed by each elevational type at each garden. By selecting a day predicted from the previous year’s flowering data to be shortly before flowering at each garden, our snapshot is one capturing gas exchange before a key developmental phase for seed production. Since gas exchange in plants is a dynamic and responsive process subject to multiple sources of feedback, and these measurements were instantaneous by nature, our work provides no information about how these traits vary throughout the season.

Yet any plasticity measured in these traits may not have necessarily resulted in the maintenance of fitness (e.g., grain weight per reproductive individual or grain weight per emerged seedling across environments). Thus, we might expect both adaptive and non-adaptive plasticity of traits to be at work. For instance, we observed a crossing of reaction norms indicative of a G x E effect for stomatal conductance, with conductance converging in the midland elevations but diverging at the lowland and highland gardens. In the highland garden, the highland type had higher conductance than types from the midlands or lowlands. This contrast may indicate that, compared to nonlocal types, stomata from highland types could more efficiently achieve greater gas exchange at cool temperatures with similar stomatal densities. On the other hand, it may indicate an inability of the lowland type to shift its stomatal conductance to the optimal phenotype favored by the local type, perhaps due to a constraint on plasticity. By contrast, the stomatal conductance of the highland type fell at lower elevations, matching the local type in the midland garden (a form of adaptive plasticity), and had significantly lower stomatal conductance than the local type in the lowland garden, indicating non-adaptive plasticity [57]. Similarly, photosynthetic rate was lowest for the least-local types at our highest and lowest elevations, perhaps indicating an inability to maintain high enough levels of this crucial function under new and stressful conditions. Additional constraints on adaptive plasticity in the highland type were revealed under the hot conditions at lower elevations, where the local type had the highest relative growth rate. These kinds of responses may have contributed to observed declines in fitness of lowland types under highland conditions and of highland types under lowland conditions.

Growth rates can be subject to selection by their local environment, with rapid growth favored, for example, when early maturity is required due to environmental constraints, such as the annual advent of reduced precipitation or cold temperatures [58, 59]. Vegetative growth and primary metabolism of cultivars–especially in maize–are known to vary, with some of this variation postulated to partially explain heterosis [60]. The plasticity in relative growth rates we noted during the year 1 growing season, with increases in growth as garden elevation decreased, may be due to higher temperatures at low elevation stimulating early season growth. Yet as water loss and carbon fixation co-occur, plants must balance the trade-offs between photosynthesis and stomatal conductance [61]. Here, we found that that selection may be operating to increase RGR across gardens and types, but that this effect may vary by year or location. Interestingly, despite increases in RGR as elevation declined, this did not translate to higher seed production in the lowland garden. In fact, seed production was highest in the cooler highland environment where plants had a longer season. Higher temperatures in the lowland garden may have been beneficial early in the season for vegetative growth, especially when rains were frequent in year 1 but became detrimental to grain fill as precipitation naturally declined and elevated temperature became heat stress, rather than a driver of rapid transpiration [41, 62]. Thus, selective pressures for this trait may vary by environment, year to year, and may only be acting and detectable during discrete growth phases.

### Implications for climate change

Our maize landraces were not equally adapted to all conditions found across the landscape, with highland and midland types especially sensitive to the warmer lowland conditions that may become more common at higher elevations with climate change. As climate change causes temperatures to increase, midland and lowland conditions can be expected to expand while previously cool highland areas will shrink [63]—a scenario which may endanger highland landraces [33]. Landrace maize populations will be forced to evolve or be discarded by the farmers who depend on them, potentially resulting in extinction [21]. Adopting and experimenting with landraces from lower elevations or similar elevations with different abiotic conditions (e.g., drier [64]) could help farmers adapt to climate change. Highland Mexican maize accounts for 18% of Mexico’s maize cultivation area [63], meaning many farmers are likely to require heat-adapted seed from lower elevations if they expect to maintain yields of previous growing seasons [65].

Yet our data suggest that, while in some years’ midland seed will perform comparably to, or even better than, highland landraces under highland conditions, these yields are not guaranteed each year. Crosses between highland and midland germplasm may produce combinations that could possibly maintain yields. Nevertheless, our data indicates that highland maize cultivars face the threat of being discarded by farmers as rising temperatures create unfavorable conditions for producing grain [21]. Likely, several years of poor performance will precipitate the loss of highland landraces in localized areas before they are completely discarded.

## Supporting information

S1 Fig Epidermal cell densities garden by elevation type. (TIF)

S1 Table Maize landrace collections from 2009. Collections were planted in 2011 and 2012 in three common gardens. Shown are elevation type, population ID numbers, collection elevations, municipal zones, and collection locations in Chiapas, Mexico. Race designations conform to Wellhausen et al. (1952). Pop. ID = population identification number.
(PDF)

S2 Table Physiological measures with units and equations making explicit their relationship to each other. (PDF)

S3 Table Destructive harvest schedule. Harvests were conducted in two field seasons, year 1 and year 2. Days after planting (DAP) and the garden average whole number of mature maize leaves (V stage) are shown for each harvest date in both years. Harvest dates were not constant across gardens due to unequal growth seasons, but vegetative stage was comparable for each harvest. In year 1, an ‘x’ marks missing harvests.
(PDF)

S4 Table Pearson correlations for year 1 fitness and physiological variables. Sample sizes ranged from n = 31–36.
(PDF)
