# Supplementary figures and images for: Physiological traits contribute to growth and adaptation of Mexican maize landraces
Source: PLoS One. 2024 Feb 1;19(2):e0290815. doi: 10.1371/journal.pone.0290815 (PMC10833551; doi:10.1371/journal.pone.0290815)

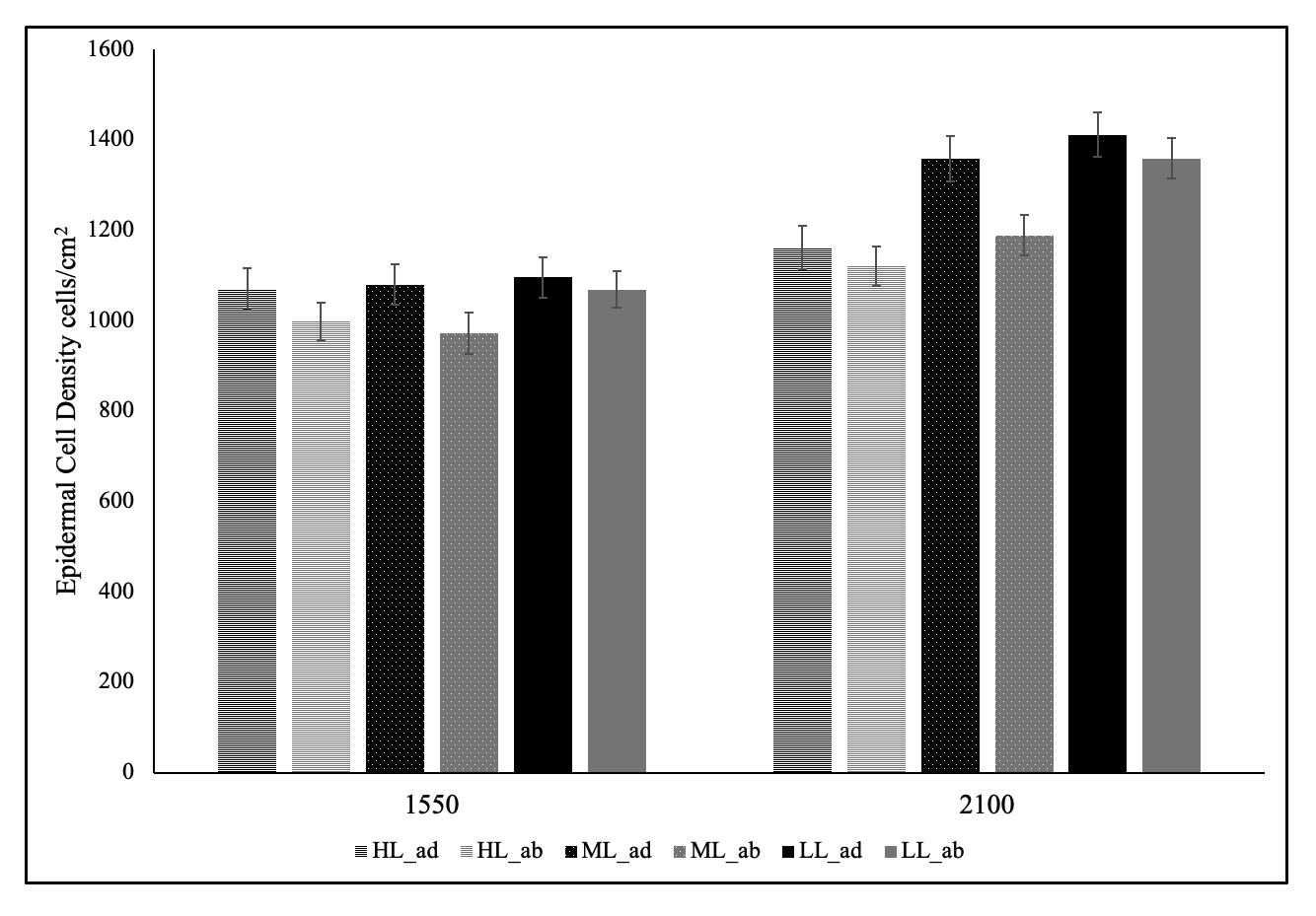

Supplement: S1 Fig — (TIF) [file pone.0290815.s001.tif]
