# Supplementary material for: Physiological traits contribute to growth and adaptation of Mexican maize landraces
Source: PLoS One. 2024 Feb 1;19(2):e0290815. doi: 10.1371/journal.pone.0290815 (PMC10833551; doi:10.1371/journal.pone.0290815)
Supplement: S1 Table — Collections were planted in 2011 and 2012 in three common gardens. Shown are elevation type, population ID numbers, collection elevations, municipal zones, and collection locations in Chiapas, Mexico. Race designations conform to Wellhausen et al. (1952). Pop. ID = population identification number. (PDF) [file pone.0290815.s002.pdf]

S1 Table. Maize landrace collections from 2009. Collections were planted in 2011 and 2012 in three common gardens. Shown are elevation type, population ID numbers, collection elevations, municipal zones, and collection locations in Chiapas, Mexico. Race designations conform to Wellhausen et al. (1952). Pop. ID = population identification number.

Elevation Type	Pop. ID	Elevation (m)	Municipality	Latitude	Longitude	Race
Lowland	1	647.81	Chicomuselo	15.89	-92.25	Tuxpeño
Lowland	4	563.21	Frontera Comalapa	15.82	-92.20	Tuxpeño
Lowland	7	598.30	La Trinitaria	15.86	-91.94	Tuxpeño
Lowland	9	594.94	La Trinitaria	15.84	-91.94	Tuxpeño
Midland	10	1532.94	La Trinitaria	16.08	-91.75	Comiteco
Midland	12	1523.80	La Trinitaria	16.11	-91.78	Comiteco
Midland	13	1583.89	Comitán de Domínguez	16.28	-92.14	Comiteco
Midland	17	1531.25	Las Margaritas	16.35	-91.92	Comiteco
Highland	20	2089.30	Comitán de Domínguez	16.36	-92.18	Olotillo
Highland	26	2153.46	San Cristobal de Las Casas	16.67	-92.65	Olotón
Highland	29	1940.77	Teopisca	16.60	-92.57	Olotón
Highland	30	2060.22	Teopisca	16.60	-92.56	Olotón
