# Supplementary material for: Physiological traits contribute to growth and adaptation of Mexican maize landraces
Source: PLoS One. 2024 Feb 1;19(2):e0290815. doi: 10.1371/journal.pone.0290815 (PMC10833551; doi:10.1371/journal.pone.0290815)
Supplement: S2 Table — (PDF) [file pone.0290815.s003.pdf]

S2 Table. Destructive harvest schedule. Harvests were conducted in two field seasons, year 1 and year 2. Days after planting (DAP) and the garden average whole number of mature maize leaves (V stage) are shown for each harvest date in both years. Harvest dates were not constant across gardens due to unequal growth seasons, but vegetative stage was comparable for each harvest. In year 1, an 'x' marks missing harvests.

	**Year 1**					
	**600 m**		**1550 m**		**2050 m**	
**Planted**	29-Jul		10-Jun		25-May	
**Harvest**	**DAP**	**V stage**	**DAP**	**V stage**	**DAP**	**V stage**
1	18	4	23	4	34	4
2	x	x	35	5	56	5
3	40	11	48	6	77	7
4	61	13	78	14	x	x
5	131	Harvest	170	Harvest	198	Harvest
	**Year 2**					
	**600 m**		**1550 m**		**2050 m**	
**Planted**	10-Aug		8-Jun		26-May	
**Harvest**	**DAP**	**V stage**	**DAP**	**V stage**	**DAP**	**V stage**
1	20	6	41	5	43	5
2	32	9	55	9	76	9
3	60	16	91	15	140	16
4	103	Harvest	165	Harvest	175	Harvest
