# Supplementary material for: Physiological traits contribute to growth and adaptation of Mexican maize landraces
Source: PLoS One. 2024 Feb 1;19(2):e0290815. doi: 10.1371/journal.pone.0290815 (PMC10833551; doi:10.1371/journal.pone.0290815)
Supplement: S3 Table — Harvests were conducted in two field seasons, year 1 and year 2. Days after planting (DAP) and the garden average whole number of mature maize leaves (V stage) are shown for each harvest date in both years. Harvest dates were not constant across gardens due to unequal growth seasons, but vegetative stage was comparable for each harvest. In year 1, an ‘x’ marks missing harvests. (PDF) [file pone.0290815.s004.pdf]

S3 Table. Physiological measures with units and equations making explicit their relationship to each other.

Abbreviation	Name	Units	Equations
A	Photosynthetic Rate	μmol m^{-2} s^{-1}	-
T	Transpiration Rate	mol m^{-2} s^{-1}	-
gs	Stomata Conductance	mol m^{-2} s^{-1}	-
PPFD	Photosynthetic Photon Flux Density	mol m^{-2} s^{-1}	-
WUE_{ins}	Water Use Efficiency (instantaneous)	mmol CO_2/mol H_20	A/T
WUE_{int}	Water Use Efficiency (intrinsic)	mmol CO_2/mol H_20	A/gs
LUE	Light Use Efficiency	mmol CO_2/ mol $m^{-2}s^{-1}$ photon	A/PPFD
RGR	Relative Growth Rate	$gg^{-1}d^{-1}$	($ln wt_2$-$ln wt_1$/t_2-t_1)
