# Supplementary material for: Physiological traits contribute to growth and adaptation of Mexican maize landraces
Source: PLoS One. 2024 Feb 1;19(2):e0290815. doi: 10.1371/journal.pone.0290815 (PMC10833551; doi:10.1371/journal.pone.0290815)
Supplement: S4 Table — Sample sizes ranged from n = 31–36. (PDF) [file pone.0290815.s005.pdf]

Supplementary Table 4. Pearson Correlations for year 1 fitness and physiological variables. Sample sizes ranged from n = 31-36

**Pearson Correlation Coefficients**								
**Prob > |r| under H0: Rho=0**								
	**H1**	**H3**	**A**	**T**	**gs**	**LUE**	**RGR**	**FIT**
**H1**	1	0.2288	0.1948	0.3134	-0.2301	0.1414	0.4038	0.2047
	0.2288	0.1861	0.2621	0.0668	0.1836	0.4179	**0.0219**	0.2531
**H3**	0.1861	1	-0.3057	-0.5120	-0.5602	-0.0694	-0.4567	-0.453
	0.1948	-0.3057	0.0698	**0.0014**	**0.0004**	0.6876	**0.0086**	**0.0072**
**A**	0.2621	0.0698	1	0.8345	0.7891	-0.0452	0.2432	0.5940
	0.3134	-0.5120	0.8345	**<.0001**	**<.0001**	0.7936	0.1798	**0.0002**
**T**	0.0668	0.0014	<.0001	1	0.6784	**0.0349**	0.4268	0.6841
	-0.2301	-0.5603	0.7890	0.6784	<.0001	0.8399	**0.0149**	**<.0001**
**gs**	0.1836	0.0004	<.0001	<.0001	1	-0.1075	0.2062	0.6231
	0.1414	-0.0694	-0.0452	0.0349	-0.1075	0.5324	0.2576	**<.0001**
**LUE**	0.4179	0.6876	0.7936	0.8399	0.5324	1	0.2878	0.3116
	0.4038	-0.4567	0.2432	0.4268	0.2062	0.2878	0.1101	0.0728
**RGR**	0.0219	**0.0086**	0.1798	0.0149	0.2576	0.1101	1	0.5784
	0.2047	-0.4529	0.5940	0.6841	0.6231	0.3116	0.5784	**0.0007**
**FIT**	0.2531	**0.0072**	**0.0002**	**<.0001**	**<.0001**	0.0728	**0.0007**	1
